# Smart Energy Management for Residential PV Microgrids: ESP32-Based Indirect Control of Commercial Inverters for Enhanced Flexibility

**DOI:** 10.3390/s25216595

**Published:** 2025-10-26

**Authors:** Miguel Tradacete-Ágreda, Alfonso Sánchez-Pérez, Carlos Santos-Pérez, Pablo José Hueros-Barrios, Francisco Javier Rodríguez-Sánchez, Jorge Espolio-Maestro

**Affiliations:** 1 Department of Electronics, University of Alcalá, 28805 Alcalá de Henares, Community of Madrid, Spain; 2 Department of Signal Theory and Communications, University of Alcalá, 28805 Alcalá de Henares, Community of Madrid, Spain

**Keywords:** energy management system, photovoltaic, prosumer, Modbus, Battery Energy Storage System, flexibility markets, wattmeter emulation

## Abstract

This article introduces a cost-effective, IoT-enabled flexible energy management system (EMS) for residential photovoltaic (PV) microgrids with battery storage, implemented on an ESP32 microcontroller. The proposed system achieves indirect control over commercial household inverters by altering wattmeter readings and utilizing Modbus communication, thereby avoiding expensive hardware modifications. A significant contribution of this work is enabling the injection of energy from the Battery Energy Storage System (BESS) into the grid, a capability often restricted by commercial inverters. Real-world experimentation validated robust performance of the proposed system, demonstrating its ability to dynamically manage energy flows, achieve minimal tracking errors, and optimize energy usage in response to both flexibility market signals and electricity prices. This approach provides a practical and accessible solution for prosumers to actively participate in energy trading and flexibility markets using widely available technology.

## 1. Introduction

The member states of the European Union are increasingly emphasizing the transition to a decarbonized energy model as a strategic priority. In this regard, significant commitments have been made to reduce greenhouse gas emissions, which necessitate substantial transformations in urban environments and energy consumption patterns [1].

In this context, electrification emerges as a key strategy for transforming the energy model of cities. Electrifying thermal demand through heat pumps and adopting electric vehicles are two fundamental changes expected in the coming decades [2,3]. These technologies offer notable advantages, such as reducing emissions in densely populated areas and achieving greater efficiency compared to traditional options [4]. However, their implementation entails a significant increase in electricity consumption and the need to adapt existing infrastructure to prevent congestion and ensure supply stability [2,3].

To address these challenges, urban self-consumption is being promoted in residential, commercial, and industrial areas. However, self-consumption installations face challenges related to the massive integration of renewable energies at the local level, such as managing energy spillage. To efficiently tackle this issue, it is proposed that these installations participate in local flexibility markets promoted by the Iberian Market Operator (OMI). These markets offer a solution to distribution network congestion and create incentives for users to optimize their investments in managing electricity generation and consumption.

The challenges and opportunities of flexibility markets and their implications for operational planning have been analyzed in recent studies [5]. One of the main challenges is the transformation of self-consumption installations from passive agents to active participants within the electrical system, requiring direct coordination with the Distribution System Operator (DSO). This demands the development of intelligent infrastructure and communication systems within the installations, as well as the implementation of an energy management system (EMS) that maximizes their economic and technical capabilities while ensuring reliability, safety, and efficiency [6].

In this new context, self-consumption installations wishing to inject energy into the grid face the following challenges: (i) the need to improve the controllability of the system as a whole through a hierarchical control scheme, essential for regulating voltage, controlling frequency, and balancing load, generation, and system operator directives [7]; (ii) the communication infrastructure must ensure connectivity to enhance system observability and facilitate or provide proper fault management through real-time state estimation of the system [8]; (iii) local coordination between the various installations comprising the system and the distribution network, including the management of energy sales to the grid and participation in the DSO’s adjustment services [6]; (iv) the integration of renewable energy sources, which are unpredictable and weather-dependent, and whose output power can change rapidly, posing a challenge to maintaining the stability of the overall electrical system [9], especially in the specific case of islands; (v) to achieve efficient energy management and significantly reduce energy spillage, it is necessary to clearly identify and quantify energy exchange at different levels, either between the various installations within our system or with the system operator [10]; and (vi) the management of numerous loads, many of which are uncontrollable, including new intelligent loads such as electric vehicles (EVs), electrolyzers, and storage systems [11].

Amid this extensive problem, this work addresses the management of a plant with photovoltaic (PV) renewable generation and storage. It proposes a low-cost, IoT-enabled Flexible EMS implemented on an ESP32 microcontroller that responds to dynamic flexibility market signals. This system uniquely achieves indirect control over commercial household inverters through wattmeter emulation and Modbus communication, thus avoiding costly hardware modifications. It is also designed to operate autonomously during periods when following aggregator signals is unnecessary, optimizing the economic benefit locally of the plant by integrating real-time electricity prices. The system seamlessly transitions between a flexibility market mode and a Voluntary Price for Small Consumers (PVPC) self-consumption mode to maximize profitability for prosumers.

### 1.1. Evolution of Energy Management Strategies in PV Microgrids

The efficient management of energy within PV microgrids, especially those integrating Battery Energy Storage Systems (BESS), is dominant for maximizing self-consumption, ensuring grid stability, and enabling participation in prospering energy markets. Extensive research has been dedicated to developing sophisticated EMS algorithms aimed at optimizing various objectives, including economic dispatch, efficiency enhancement, and improved system stability [12,13,14]. These algorithms often leverage mathematical optimization techniques such as Mixed-Integer Linear Programming (MILP) and Markov Decision Processes (MDPs) to achieve optimal day-ahead dispatch, peak shaving, and power reduction strategies [12,14]. The integration of BESS is consistently highlighted as a critical component for providing flexibility, allowing for energy trading, and supporting grid services [13]. Recent advancements in EMS research have increasingly incorporated artificial intelligence (AI) and machine learning (ML) techniques to address the inherent uncertainties of renewable energy sources and dynamic load profiles [15]. AI-driven approaches, including reinforcement learning, neural networks, and various meta-heuristics (e.g., Particle Swarm Optimization (PSO), Genetic Algorithms (GAs)), are employed for robust forecasting, optimal decision-making under uncertainty, and anomaly detection [15]. These intelligent algorithms contribute significantly to enhancing the resilience and economic viability of PV microgrids by adapting to real-time conditions and optimizing energy flow. Furthermore, the expansion of distributed energy resources has emphasized the critical role of Demand-Side Management (DSM) and demand response (DR) programs within EMS frameworks [16]. BESS plays a pivotal role in enabling active participation in DR initiatives, which range from price-based programs (e.g., Time-of-Use (ToU), Critical Peak Pricing (CPP), Real-Time Pricing (RTP)) to incentive-based schemes like load shifting, valley filling, and peak clipping [16]. These strategies not only benefit prosumers by reducing energy costs but also contribute to grid stability by alleviating congestion and reducing peak demand. The growing interest in vehicle-to-grid (V2G) and vehicle-to-home (V2H) capabilities also positions electric vehicles (EVs) as flexible loads and mobile energy storage units, further complicating and enriching EMS objectives [15]. Finally, recent research in EMS for PV microgrids focuses heavily on advanced control and optimization techniques [17]. One notable development is the use of intelligent algorithms, such as the Sparrow Search Algorithm (SSA), to fine-tune Proportional–Integral (PI) controllers. The SSA-tuned PI controller is particularly effective at optimizing dynamic performance, power quality (e.g., reducing Total Harmonic Distortion (THD)), and energy extraction through precise Maximum Power Point Tracking (MPPT). This intelligent approach has demonstrated superior results in simulations, showing a faster response time and higher active power output compared to conventional controllers, making it highly suitable for robust, real-time smart grid integration under varying conditions.

### 1.2. Limitations of Current Real-World Implementations

Despite the proliferation of advanced EMS algorithms in academic literature, a notable and persistent limitation remains the lack of studies demonstrating comprehensive real-world validation in physical microgrids. The majority of innovative strategies are primarily verified through simulations or emulations, which, while valuable for theoretical exploration, often fall short in addressing the practical complexities and uncertainties of real-world deployment. Existing real-world tests often focus on isolated aspects such as monitoring or localized control of specific microgrid elements [18,19,20,21]. When comprehensive flexibility strategies are implemented in physical testbeds, they frequently rely on custom-developed or industrial-grade inverters [22]. For instance, large-scale research facilities like those at the National Renewable Energy Laboratory (NREL) engage in extensive Power Hardware-in-the-Loop (PHIL) testing and microgrid deployment using specialized inverter hardware [22]. While crucial for advancing grid integration, these approaches do not directly translate to the challenges faced by typical residential or small-scale prosumers utilizing commercially available household-grade inverters.

The following revised paper [12], for example, proposes an EMS for a microgrid with DER and a BESS. The system is designed to optimize the economic dispatch of resources with a day-ahead approach using a MILP model. The EMS employs a simple logic and a microservices architecture, which includes a database, a graphical user interface (GUI), and an API, allowing for real-time remote monitoring and control. The system was tested through simulations and emulation, validating its performance under realistic conditions in both grid-connected and isolated modes.

Moreover, in another study [13], an innovative stochastic energy management strategy is presented for autonomous PV microgrid systems in environments with unpredictable energy consumption. The strategy uses an MDP to model the random variability of load consumption, enabling real-time optimization of PV generation and the charging and discharging of batteries or BESS. The tests were conducted through simulation under different weather and battery load conditions, demonstrating improved system efficiency and microgrid stability.

Additionally, ref. [14] investigates the economic opportunities of power reduction and peak shaving in residential systems that combine solar PV and battery storage (BESS). The conditions under which prosumers would find participation in ancillary services markets in low-voltage networks attractive are analyzed. The results indicate that these strategies can be beneficial, especially peak shaving, which extends the battery lifespan, although power reduction has a limited impact on the levelized cost of energy. Like the other studies, this one also conducts verification tests through simulation.

As can be seen, multiple studies propose flexibility strategies for small microgrids or domestic prosumer installations, where the injection of energy from the battery or BESS into the grid is crucial. All these works introduce innovative EMS strategies that would not be possible without the active and bidirectional role of the BESS. However, it is observed that all the proposals in the state of the art conduct verification tests through simulation or emulation of all the involved systems, without utilizing real physical microgrids or on-site experimental setups for validation.

On the other hand, there are many studies that conduct real-world tests on microgrids, but almost all of them focus solely on the monitoring of system components and, in some cases, on the localized and isolated control of a particular element. In [18], a SCADA system is designed to monitor a DER microgrid based on an ESP32. Similarly, in [23], a SCADA system for a DER microgrid is designed using the widely adopted IEC61850 standard for electrical distribution networks. Additionally, in [19], a SCADA system is implemented for a PV installation with a weather station, uploading all data to the Thingspeak cloud. Finally, one study [20] monitors and controls a microgrid with grid injection by applying a flexibility strategy, but it uses an emulated distributed generator and an industrial-grade inverter.

### 1.3. The Emergence of Smart Inverters and Direct Control

Commercially available household inverters have traditionally presented significant limitations concerning precise, dynamic, and bidirectional power flow control for participation in flexibility markets. These devices are often designed for simplified grid-tied operation, primarily focused on injecting PV energy into the grid while typically restricting or complicating the injection of stored battery energy back to the grid in a controlled manner, or responding to external grid signals. This necessitates costly hardware modifications to the inverter itself to enable such functionalities. Recent technological advancements have introduced “smart inverters” into the market, which are increasingly equipped with direct communication interfaces, such as Modbus (both RTU and TCP) and SunSpec protocols [24,25]. Leading manufacturers like SMA and SolarEdge now offer these capabilities, allowing for direct monitoring and, crucially, direct control over various parameters, including active power limitation setpoints and reactive power compensation [24,25]. This represents a significant step towards enabling residential PV and battery systems to actively participate in grid-support functions and flexibility services, potentially mitigating the need for complex indirect control methods. However, the full utilization of these direct control capabilities can sometimes be constrained by manufacturer-specific access protocols (e.g., requiring special passwords for certain advanced settings) or may not be present in the vast installed base of older or less sophisticated commercial inverters.

### 1.4. Research Gaps and Contributions of the Authors

Despite numerous innovative strategies for EMS in microgrids, a significant gap exists in their real-world validation. Most research relies on simulations and emulations, which, while insightful, often fail to capture the complexities of actual microgrids. Specifically, current studies frequently focus on energy management algorithms that primarily involve injecting surplus PV energy into the grid and using BESS for energy trading. Furthermore, the limited real-world experiments tend to utilize custom-developed or industrial-grade inverters, which are not representative of standard residential setups. This leaves a critical gap in the literature concerning the practical implementation of these strategies with commercially available household-grade inverters.

Given these challenges and the diverse range of installed inverters, indirect control methods for commercial inverters are crucial. These methods offer a cost-effective way to unlock the flexibility of existing residential PV installations without requiring expensive hardware modifications. While not extensively documented with real-world validation in academic literature, these pragmatic solutions are key to integrating “non-smart” inverters into advanced energy management schemes.

Therefore, this work addresses a critical gap by implementing a low-cost system that enables any commercially available household inverter to inject not only excess PV energy but also energy from the battery, overcoming an imposed limitation that these inverters typically have. Thus, the charging and discharging of the BESS from and to the grid can be precisely controlled at any desired time resolution. Therefore, this system allows for the true injection of energy from the batteries of a domestic prosumer installation, using a standard commercial household inverter currently available on the market. This approach eliminates the need to develop custom inverters or rely on industrial-grade inverters, making it more practical and accessible for residential users. By unlocking the full potential of household inverters, this contribution paves the way for prosumers to actively participate in energy trading and flexibility markets, using cost-effective and widely available technology.

## 2. Description of the Framework

This section first addresses the main problem of the limited flexibility offered by commercial inverters to residential PV systems, as well as the integration of the proposed solution within the flexibility market framework. Subsequently, the utilized microgrid and all its elements for implementing said solution are detailed, also presenting the final configuration of the microgrid.

### 2.1. Problem Overview

One of the main challenges in managing energy within PV microgrids is the limited control over power flows in real time. In conventional systems, inverters often lack the capability to dynamically adjust power injection or absorption directly in response to the needs of the grid or market signals. Moreover, commercial residential inverters typically prohibit the injection of energy from the BESS into the grid, limiting the use of stored energy solely to local self-consumption. This restricts the ability to optimize energy usage, balance supply and demand, and participate effectively in demand flexibility services. Without real-time control, excess energy may go unused, and opportunities for energy trading or cost-saving strategies, such as peak shaving, are missed. Additionally, this limitation can reduce the overall efficiency and financial viability of renewable energy systems, particularly in residential prosumer setups.

To overcome this, our solution employs a flexible control strategy based on the ESP32 microcontroller, which allows for indirect influence on the behavior of the inverter through the manipulation of external wattmeter readings. By emulating these readings, the system can effectively control the amount of energy injected or absorbed by the inverter, thereby optimizing power flows in real time according to market conditions and grid demands. This innovative approach enables a higher level of integration with demand response programs and flexibility services without requiring hardware modifications to the inverter itself, making it a cost-effective and scalable solution for residential and small-scale energy systems.

**Figure 1:**
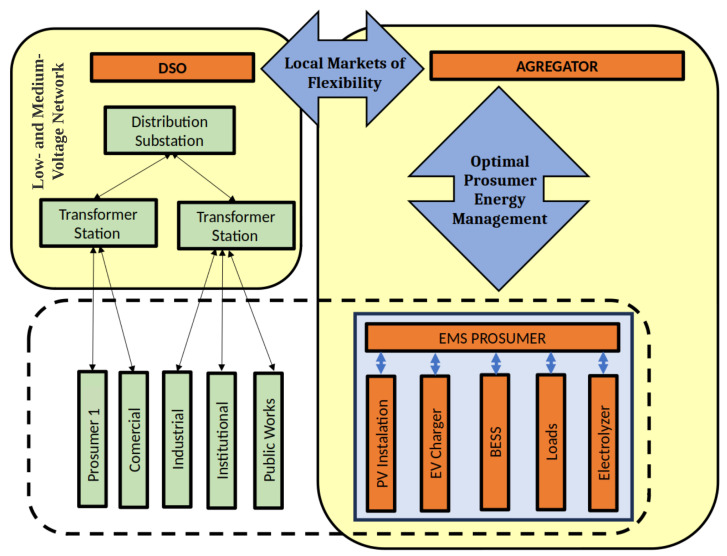
Schematic of the low- and medium-voltage network highlighting the roles of the DSO and an aggregator in coordinating distributed energy resources. The aggregator serves as a key link between the DSO and individual prosumer EMS, which oversee local assets such as PV installations, EV chargers, BESS, loads, and electrolyzers.

The aggregator, an entity responsible for consolidating the DER (such as PV generation and battery storage) from various prosumers to offer collective flexibility services in energy markets, will receive signals based on the local conditions of the distribution network. In this way, it will be able to send flexibility instructions to each of the downstream users, who must follow these instructions at all times. These flexibility signals will be generated considering the specific availability of each user participating in this market, providing them with an economic benefit in return. Figure 1 shows the general architecture of this distribution network.

This work emulates the behavior of these signals under the criterion that, as PV energy production increases, the network becomes more congested. Therefore, the flexibility signals act in the opposite direction, incentivizing congestion reduction. However, this study does not address the design of a specific market; instead, it demonstrates the ability to follow the proposed functionality effectively in a real plant.

### 2.2. Microgrid Description

**Figure 2:**
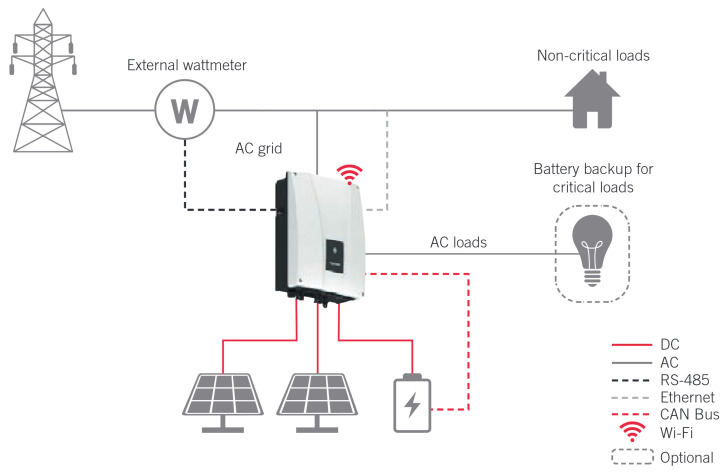
Previous connection diagram between elements of the microgrid used.

The experimental microgrid comprises essential components for residential PV self-consumption with battery storage or BESS. Energy generation is provided by nine PV panels, offering a total power of 3 kW. A BESS with a 7.1 kWh capacity is used for energy storage. The core of the system management is handled by an inverter, which converts DC to AC power and controls charging and discharging processes of the BESS. Loads in the microgrid are segregated: critical loads are connected to a dedicated port on the inverter for an uninterrupted power supply, while non-critical loads are connected externally to the AC grid. The inverter manufacturer allows control over these non-critical loads via an external wattmeter that monitors the power flow between the grid and the microgrid. For this control system, an external wattmeter is specifically used to monitor power exchange with the grid. This microgrid configuration (shown in Figure 2), combining PV generation, the BESS, the inverter, and controlled loads, establishes the platform for implementing the flexible energy management system.

Furthermore, the proposed system utilizes the ESP32 microcontroller [26] as the central IoT-enabled control unit, relying on its dual Modbus capabilities to manage the microgrid. Specifically, the ESP32 employs Modbus RTU over an RS-485 physical layer in two ways: operating simultaneously in master mode to read data from the actual external wattmeter, and in slave mode to emulate the wattmeter and send manipulated readings to the inverter. Furthermore, the system uses Modbus TCP/IP over Wi-Fi to retrieve essential operational data, such as the battery state of charge (SoC), directly from the commercial inverter, allowing for comprehensive energy management decisions.

### 2.3. Control Scheme

**Figure 3:**
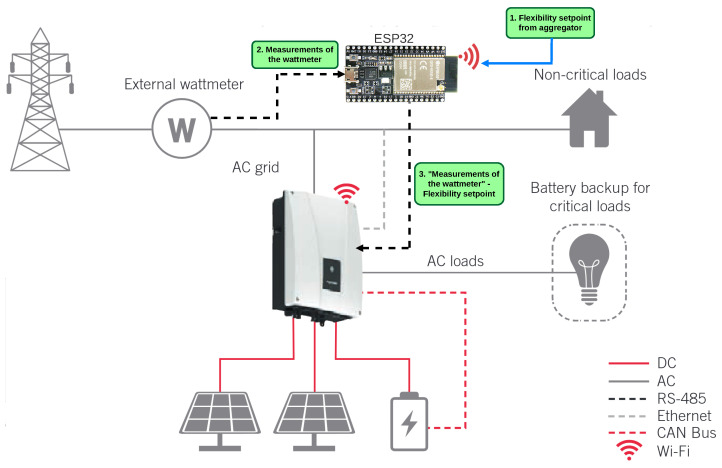
General system and control operation diagram. This control strategy outlines the interaction between all components, including the ESP32, wattmeter, inverter, battery system, and the grid. This approach enables the system to optimize energy management autonomously, adapting to both internal and external conditions in real time.

Our modified control system, illustrated in Figure 3, leverages the ESP32 microcontroller to manipulate the power readings from the external wattmeter, effectively tricking the inverter into adjusting its output power in response to perceived changes in load or generation. In a typical setup, the wattmeter measures the power flow between the PV system, the loads, and the grid. The inverter relies on these measurements to regulate its power injection or absorption, ensuring balance within the system.

Then, the aim of the control system is to completely control the power flow into and from the grid. By configuring the inverter in “no surplus injection” mode, the control system guarantees that the inverter does not send excess energy into the grid in an uncontrolled manner, while still allowing the inverter to dynamically adjust its output based on the needs of the BESS and loads. The ESP32 acts as the brain of the control system, continuously monitoring and altering the wattmeter signals to achieve desired energy management objectives, such as peak shaving, cost savings, or grid support.

The control system operates as follows: (i) the ESP32 first receives a setpoint from an external aggregator to determine the desired power into or from the grid in relation to the microgrid; (ii) the ESP32 then obtains the actual power output measurements from the wattmeter; and finally, (iii) the control algorithm running on the ESP32 generates an artificial offset, which is added to the readings of the wattmeter, effectively making the ESP32 act as the wattmeter from the perspective of the inverter. This offset creates a scenario where the inverter perceives a shift in load or generation conditions, prompting it to either inject more power into the grid (by artificially adjusting for an increase in non-critical load consumption) or absorb energy from the grid (by artificially adjusting for an excess in local generation). This indirect control method enables precise management of grid power flow without requiring direct commands to the inverter, a feature not supported by many commercially available inverters.

Then, the energy flow within the system operates as follows:

- Power Injection: When the system detects a need to inject energy into the grid, the ESP32 adds a negative offset to the wattmeter readings to indicate higher demand on non-critical loads. This prompts the inverter to respond by injecting the required amount of power to balance the system, drawing either from the PV generation or the stored energy in the BESS.
- Power Absorption: Conversely, when the system needs to absorb excess energy from the grid into the battery (e.g., during periods of low demand), the ESP32 adjusts the wattmeter readings with a positive offset to simulate an overproduction of energy from the PV system. The inverter, in response, absorbs power from the grid to maintain equilibrium.

## 3. Implementation

The implementation of the control system focuses on the hardware assembly and the communication protocols required to manage the energy flows between the PV installation and the grid.

### 3.1. Hardware Setup

The microgrid consists of nine Talesun Solar panels [27] with a total capacity of 2970 W for energy generation. The energy is stored in Pylontech Force L2 lithium-ion batteries [28] with a capacity of 7.1 kWh. An Ingecon Sun Storage 1Play TL M inverter [29] manages the conversion of DC to AC and controls the battery charging and discharging process. The loads are connected to the critical load port of the inverter, ensuring uninterrupted power supply. Additionally, the manufacturer of the inverter enables the control of non-critical loads, connected externally to the AC grid, through the installation of an external wattmeter that monitors the power flow between the grid and the microgrid. For the control system, this external wattmeter will indeed be used, with the selected model being the Carlo Gavazzi EM111DINAV81XS1X [30]. These components are connected as illustrated in Figure 2, which depicts the overall system layout.

**Figure 4:**
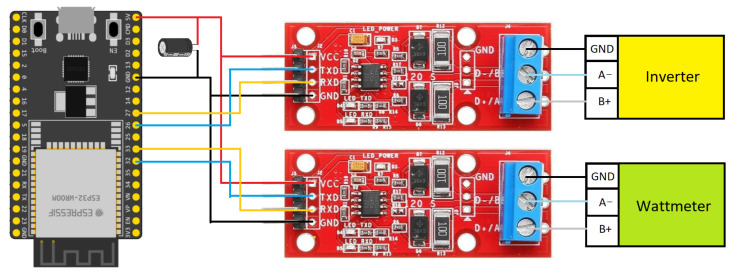
Electronic controller diagram.

**Figure 5:**
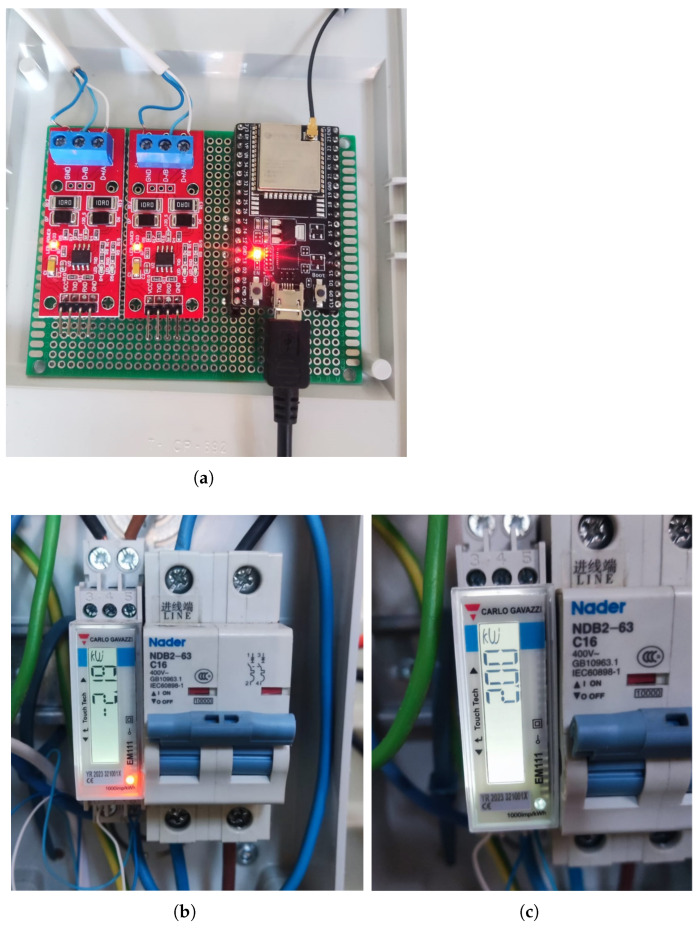
Electronic controller hardware assembly. (**a**) TTL-RS485 transceiver modules along with the ESP32 microcontroller. (**b**) External wattmeter during grid energy injection. (**c**) External wattmeter during grid energy absorption.

The ESP32 microcontroller is the core of the control system, managing all communications and decision-making, as shown in Figure 3. The hardware was mounted on a circuit board, where various components were soldered, including modules for communication via RS485 and power regulation. The system is powered by a 5V power supply through the USB of the ESP32, which also powers the TTL-RS485 transceiver [31] modules, allowing the control of Modbus communications. The described elements are shown in Figure 4, with the hardware assembly displayed in Figure 5.

A critical aspect of the hardware setup is the communication interface with the photovoltaic inverter. Two TTL-RS485 transceiver modules were utilized, each connected to a distinct UART (Universal Asynchronous Receiver–Transmitter) port on the ESP32. This dual UART configuration enabled simultaneous Modbus RTU communication:

- UART1 (Pins 26 RX, 27 TX): Configured for slave mode, this port emulates the external wattmeter, responding to requests from the inverter.
- UART2 (Pins 32 RX, 33 TX): Configured for master mode, this port communicates with the actual external wattmeter to acquire real-time data.

A notable challenge during the connection process was the inverse polarity of the Modbus terminals between the inverter and the RS485 adapter modules. While standard Modbus connections typically involve linking A to A and B to B terminals, it was necessary to cross-connect the cables (Inverter A to Module B, Inverter B to Module A) to ensure correct positive-to-positive and negative-to-negative signal alignment, thereby preventing communication errors such as bit inversion and byte loss.

### 3.2. Communication via Modbus

The control system relies on the Modbus protocol to communicate between the ESP32, inverter, and wattmeter. Two types of Modbus communication are used:

- Modbus RTU: The ESP32 acts in dual roles: as a slave, it manipulates the wattmeter readings and sends them to the inverter, indirectly controlling the power flows. Simultaneously, it functions as a master, requesting real-time power readings from the actual wattmeter to process and adjust the behavior of the system dynamically.
- Modbus TCP/IP: Over WiFi, the ESP32 retrieves critical operational data from the inverter, such as the SoC of the battery and performance metrics, to further optimize energy management decisions.

### 3.3. Wattmeter Emulation and Initial Scanning

**Figure 6:**
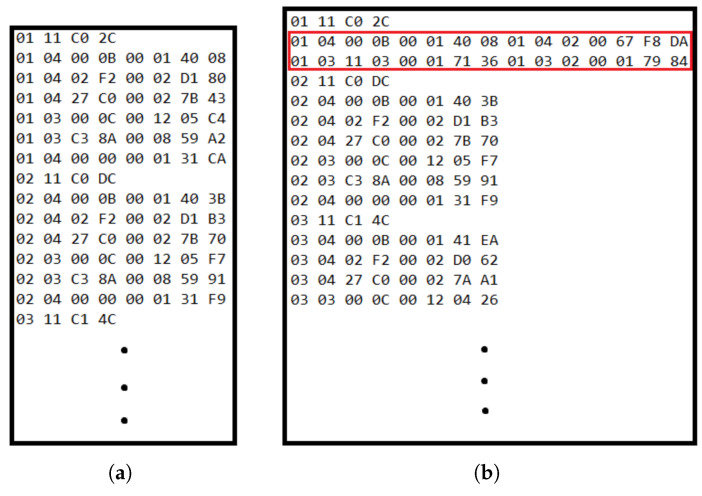
Captured Modbus data exchange between wattmeter and inverter. When analyzing the captured data, we observed a long list of exchanged messages. However, our focus was narrowed to messages addressed to slave ID 1, which corresponds to the wattmeter. These messages are easily identifiable as they begin with the hexadecimal byte “01”. From this data traffic capture, we determined that the wattmeter responds to two specific readings performed by the inverter during its initial scan. These crucial interactions are highlighted in red in Figure 6b. (**a**) External wattmeter not connected to the inverter. (**b**) External wattmeter connected to the inverter.

The fundamental control strategy relies on the ESP32 emulating an external wattmeter to influence the behavior of the inverter. This emulation process involved:

- Modbus Traffic Analysis: An initial analysis of the native Modbus traffic between the inverter and a real wattmeter revealed that the inverter primarily queries two specific registers: 30012 (for wattmeter model identification) and 404356 (for power readings). Figure 6 shows one of the tests for this initial analysis.
- Register Creation: The software of the ESP32 has been designed to create and populate these specific Modbus registers internally during startup, allowing it to accurately mimic the behavior of, namely, the wattmeter. After this initial exchange, the inverter periodically reads 16 consecutive registers starting from address 30001. These registers contain critical electrical parameters, such as active power, voltage, and current, that the inverter uses to monitor the power flow between the grid and the microgrid. The ESP32 emulates this behavior, responding with the necessary data to maintain seamless operation.
- Sampling Rate: A sampling time of 500 ms was selected for reading wattmeter data, striking a balance between data refresh rate and optimizing the processing load of the ESP32. The sampling time of 500 ms was selected because it is precisely half the period of 1 s that the inverter uses to sample the wattmeter readings. This ensures the ESP32 is always prepared with updated data before the inverter makes its request.

### 3.4. Access to Inverter Registers

Once the initial scanning process is completed, the ESP32 also communicates with the inverter via Modbus TCP/IP over WiFi. This communication allows the system to retrieve critical information such as the SoC of the battery, the energy generated by the PV panels, and other system metrics. This data can be used to optimize energy management within the microgrid.

During the TCP/IP communication setup, the ESP32 is connected to the local network and establishes a connection with the inverter at its assigned IP address via port 502, the standard port for Modbus TCP/IP. This process enables real-time access to the internal data of the inverter, divided into different zones for various system components: battery, panels, wattmeter, among others. The ESP32 alternates between reading these zones, ensuring that all relevant data is collected without overloading the system.

### 3.5. Obtaining PVPC Prices

An additional feature of the control system is its ability to retrieve PVPC electricity prices in real time from an external API of ESIOS [32]. By integrating this data, the system can optimize energy management based on market conditions, dynamically adjusting energy flows to maximize economic efficiency. The ESP32 accesses the API daily, parsing the price data and incorporating it into decision-making processes for energy injection or absorption.

### 3.6. ESP32 Software Development

The software, developed within the Arduino IDE 2.3.4 environment adapted for ESP32, is responsible for orchestrating all communication and control logic. Key aspects of the software implementation include

- Library Integration: Essential libraries, including those for Modbus communication (RTU and TCP/IP), were integrated to facilitate data exchange.
- System Initialization: The program initializes various components, including Modbus RTU (both master and slave roles), Modbus TCP/IP client, and WiFi connectivity.
- Wattmeter Emulation Logic: A dedicated function, CreateIregs(), is executed once at startup to set up the registers of the emulated wattmeter. A callback function is then assigned to the specific power reading register (404356) to trigger a flag, signaling the completion of the initial scan by the inverter and enabling the controller to begin delivering dynamic wattmeter readings.
- Data Acquisition: The software continuously reads data from the physical wattmeter (via Modbus RTU master) every 500 ms using the millis() function to ensure non-blocking operation.
- PVPC Price Acquisition: The system incorporates a function (getPVPC()) to retrieve real-time energy prices from the Spanish regulated market (Red Eléctrica Española-REE) API. This involves sending HTTP GET requests and parsing the JSON response to extract the 24-hourly prices, which are crucial for optimizing energy management strategies.

**Figure 7:**
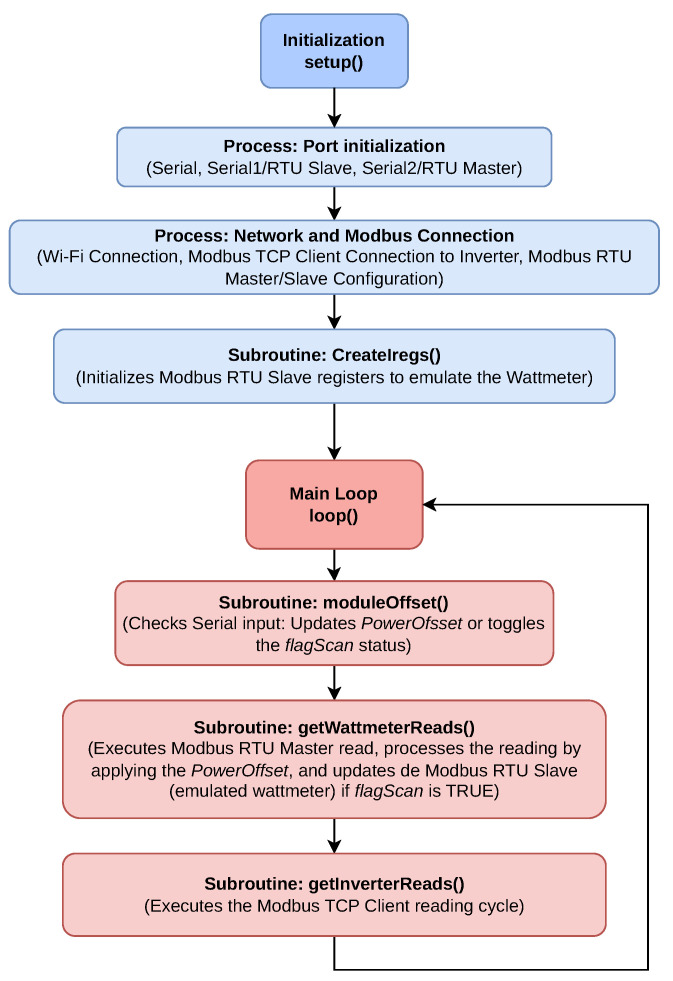
Pseudocode flowchart of the main control loop. The chart details the reading of system variables and the subsequent logic used to apply the wattmeter emulation offset, enabling precise, indirect control of the power injection and absorption of the inverter.

The complexity of handling multiple communication protocols (Modbus RTU master/slave, Modbus TCP/IP, and Wi-Fi connection for PVPC price retrieval) necessitates a clear visualization of the control logic. Figure 7 presents the pseudocode flowchart that illustrates the main sequential operation, particularly how data is acquired and processed to generate the wattmeter emulation setpoint.

### 3.7. Flexibility Framework

Finally, our system operates in two distinct modes to optimize energy management:

- Flexibility Market Mode: Firstly, during the hours when flexibility markets are active, an aggregator will send signals for tracking. In this work, these signals will not be generated directly but will be emulated, and the device must comply with them at all times.
- PVPC Self-consumption Mode: The second mode of operation applies to the hours when the flexibility market is not active. During these periods, the system will operate locally, using retail market prices (in this case, the PVPC) to develop an economic optimization strategy. This involves deciding when to charge or discharge the battery based on retail market prices. It is worth noting that these prices are published at 6:00 p.m. on the previous day, enabling the system to plan in advance the best hours of the day for these operations, provided that it is not participating in flexibility markets at that time.

The practical case will demonstrate the implementation and functionality of these two operating modes.

## 4. Results

The performance of the system was rigorously evaluated on 11 October 2024, at a testbed located in the Polytechnic School of the University of Alcalá (Spain), under two distinct operational modes. The first one, flexibility market mode, was active until 2:45 p.m., after which the second one, PVPC self-consumption mode, prevailed for the remainder of the day. The primary objective of this comprehensive assessment was to demonstrate the robust and correct functioning of the proposed system, with these specific operational strategies serving as illustrative use cases for validation. The assessment also aimed to analyze the behavior of the system under these conditions and their associated economic implications, which are detailed in the following subsections.

### 4.1. Setup

**Figure 8:**
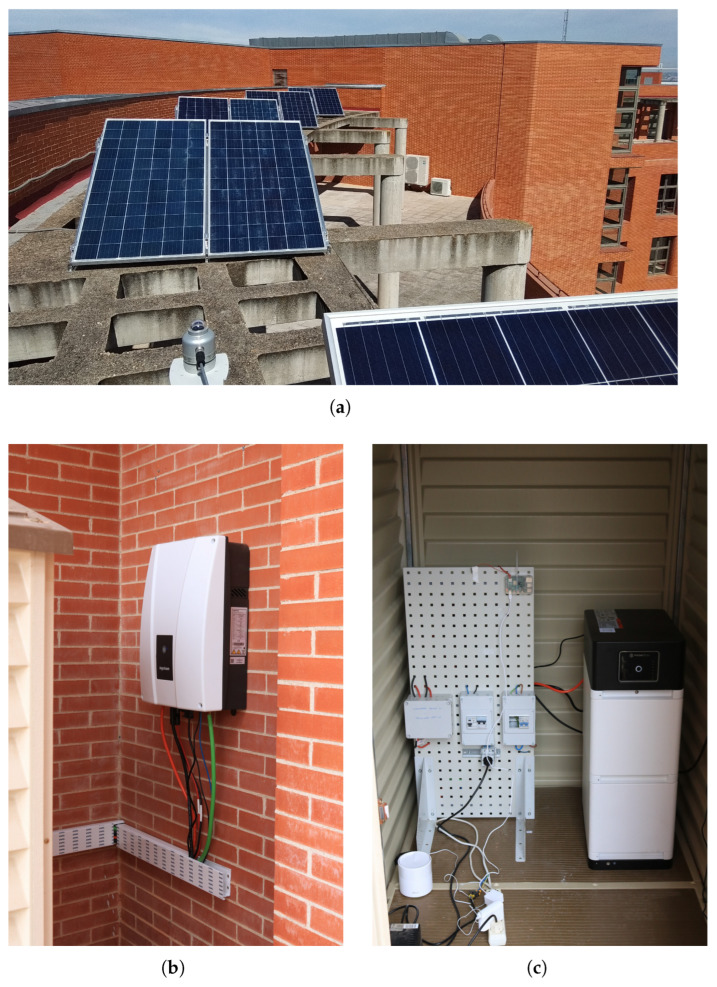
PV installation used as experimental setup. (**a**) Nine “TP672P-330W” PV panels in a 3 × 3 configuration. (**b**) “Ingeteam Ingecon SUN Storage 6TL M” inverter. (**c**) BESS “PYLONTECH L2 Force” of 7.2 kWh.

For the experimental setup, a PV installation was used with a rated load power of 3 kW on the rooftop of the Higher Polytechnic School at the University of Alcalá (UAH), Spain (Figure 8). This installation consists of 9 “TP672P-330W” PV panels [27], an “Ingeteam Ingecon SUN Storage 6TL M” inverter [29] for MPPT, a BESS “PYLONTECH L2 Force” [28] of 7.2 kWh, two variable resistive loads of 2 kW each have been used as the loads, and the control system developed in this article.

### 4.2. General System Performance

Before conducting energy flow tests, the system was connected to the PV plant to ensure that the controller, inverter, and wattmeter were properly integrated. Initial tests were performed to verify that the inverter was correctly receiving and processing the wattmeter readings, ensuring accurate control of power flows. Once the system was confirmed to be functioning correctly, further tests were carried out to evaluate the ability of the controller to manage energy injection and absorption based on real-time setpoints. These setpoints command the net power exchange between the grid and the PV microgrid.

On the one hand, in the energy injection tests, the system was given a setpoint to inject surplus energy into the grid (such as during peak electricity price periods), allowing the sale of excess energy at a favorable rate. The controller successfully manipulated the wattmeter readings, and the inverter adjusted its output accordingly to export the surplus energy to the grid. On the other hand, for the energy absorption tests, the system was instructed to absorb energy from the grid (such as during periods when electricity prices were low), emulating real-time flexibility signals. The controller dynamically adjusted the inverter to charge the batteries, accurately responding to the absorption setpoint.

**Figure 9:**
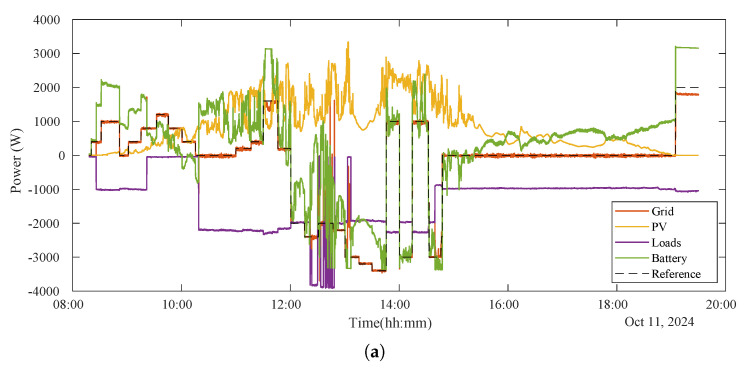

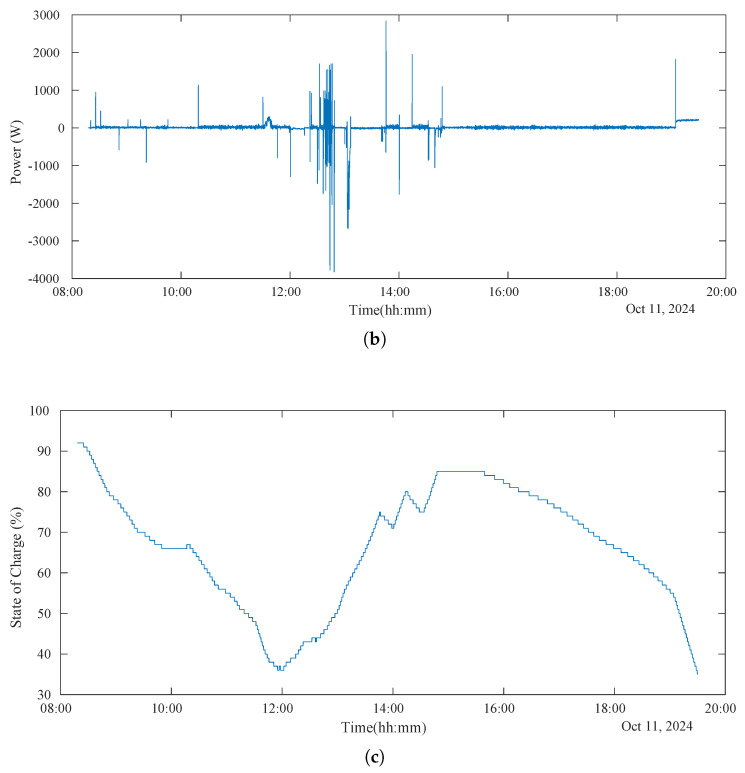
The data presented here are results from a test performed on 11 October 2024, at a testbed within the Polytechnic School of the University of Alcalá, Spain. The experiment, which spanned from roughly 8:30 a.m. to 19:30 p.m., operated under two distinct modes. Throughout the test, different setpoints were sent to the inverter, simulating commands of an aggregator to regulate power exchange with the grid. The measured variables of the PV microgrid during this trial are consequently displayed. (**a**) The graph displays all measured electrical variables of the PV microgrid during the test day. It can be observed how the setpoint commanded to the inverter (dashed black line) dictates the power flow exchanged between the PV microgrid and the grid (red line), which the inverter is confirmed to follow. The PV production and local loads, however, are independent of the setpoint. Conversely, the BESS power dynamically responds to the other variables in order to achieve the commanded setpoint. (**b**) This graph shows the power error, which is the difference between the actual power exchanged with the grid and the commanded setpoint. It can be seen that this error is generally very low or even zero. However, during transient periods when local load consumption changes or the setpoint command shifts, this error temporarily increases before stabilizing again. (**c**) This graph displays the BESS state of charge (SoC), as provided by the inverter. This variable clearly illustrates the energy consumed or stored by the BESS throughout the test, as well as how following the setpoint influences its charge level.

**Figure 10:**
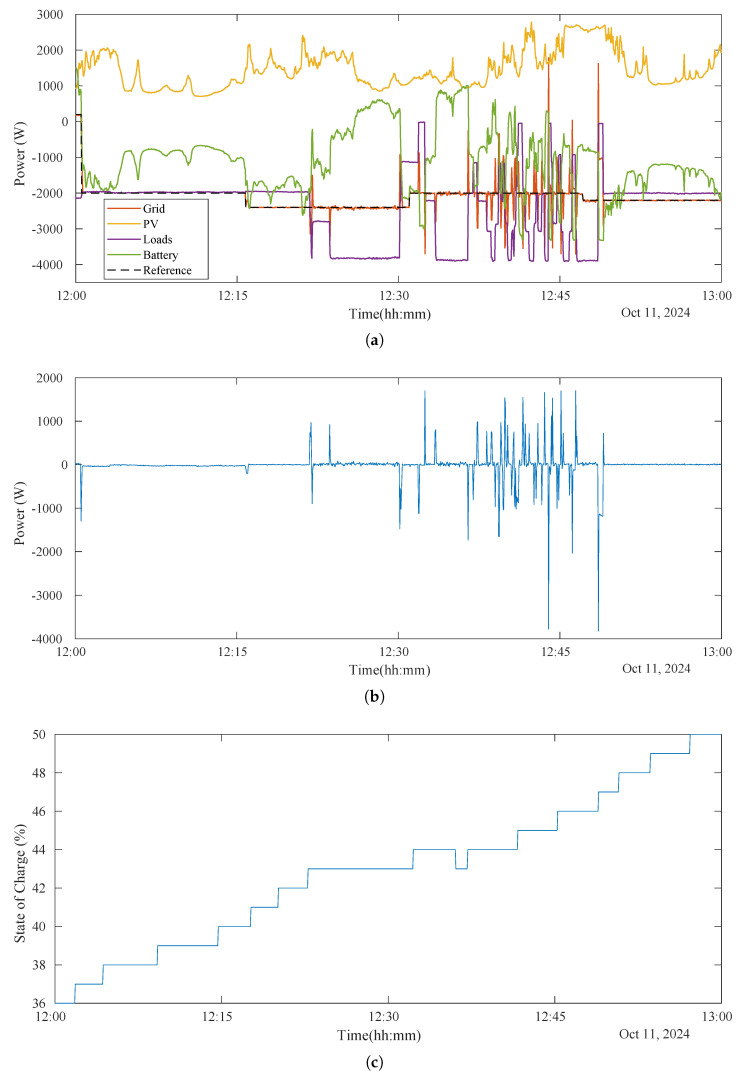
The results in these figures correspond to the same test day as Figure 9, but they are enlarged for the time range from 12:00 p.m. to 13:00 p.m. This allows for a clearer observation of specific aspects of the flexibility strategy and how the inverter responds to setpoint commands and various transients. (**a**) During the flexibility market mode, it is worth noting that the BESS was effectively utilized by being forced to charge from both PV surplus and the grid when required. This compensated for the power that variable loads could not manage. Conversely, when the inverter is compelled to inject power into the grid and there is not enough PV energy, the BESS is forced to directly supply that power to the grid. (**b**) It can be observed that the tracking error between the power exchanged with the grid and the setpoint is very low, often even zero. Only during transient periods, when local load consumption or the setpoint changes, does this tracking error show temporary peaks before stabilizing again. (**c**) In this detailed graph, it can be seen how, during this flexibility market mode, the BESS generally absorbs PV energy. However, in the middle of the period, it is forced to supply energy to local loads, pausing its charging and even discharging slightly.

In Figure 9a and Figure 10a, it can be seen how the power from and to the grid (in red) follows perfectly the reference setpoint given by the ESP32 to the inverter (in dashed black), modifying the wattmeter readings. As previously introduced, to enable the injection and absorption of energy from and into the grid from both the PV array and the BESS, the control strategy involves artificially adjusting the reported power load consumption. This compels the inverter to increase or decrease its power output, which is then subsequently injected into the grid or absorbed from it.

### 4.3. Flexibility Market Mode

During the flexibility market mode (until 14:45 p.m.), the system prioritizes consumption of PV energy and not injecting the surplus energy. For this, negative setpoints from the aggregator during periods of high PV production indicated the need to increase local consumption (clearly seen from 12:00 p.m. to 13:00 p.m. in Figure 9a and enlarged in Figure 10a). To achieve this when the variable loads were not consuming enough (when the setpoint is more negative than load consumption), the BESS was utilized effectively forcing it to charge from both the PV surplus and the grid, therefore compensating for the power that the variable loads were not able to manage (seen from 12:15 p.m. to 12:23 p.m. approximately in Figure 10a). This operational strategy ensured compliance with the directives of the emulated aggregator, contributing to the local flexibility market.

Moreover, when the setpoint matches the actual load consumption, the proposed system commands the inverter to power these loads solely with grid energy. This means any excess PV energy is then used exclusively to charge the BESS (as seen from approximately 12:00 p.m. to 12:23 p.m. in Figure 10a,c). If the BESS is already full, the inverter is forced to exit MPPT mode. Conversely, when the setpoint is set higher (more positive) than the load consumption, the inverter is prompted to maximize self-consumption, supplying the loads with both PV and BESS energy (visible from approximately 12:23 p.m.to 12:30 p.m. in Figure 10). Also, if the setpoint is positive, the inverter is forced to inject power into the grid. If there is not enough PV energy, it will draw power from the BESS (as seen from approximately 8:30 a.m. to 10:00 a.m. in Figure 9a,c, where positive battery power indicates discharging).

Finally, the graphical analysis of this period is shown in Figure 9b and Figure 10b, revealing minimal tracking errors, with the system maintaining a high degree of accuracy. The power error between the real grid power exchange and the setpoint is only non-zero during transient changes in load consumption or setpoints. Furthermore, in Figure 10, a zoomed-in view of this period is shown, which highlights the dynamic behavior of the battery, which played a critical role in maintaining energy balance. Then, it is worth noting that despite occasional transients, overall system performance remained robust. Nevertheless, during abrupt, step-type load changes, the transients observed in the setpoint tracking lasted an average of 6 s and exhibited a maximum power error peak equal to the change in the power of the load.

### 4.4. PVPC Self-Consumption Mode

Following the flexibility market period, the system transitioned to the PVPC self-consumption mode at 14:45 p.m. In this mode, the operational strategy was dictated by dynamic electricity pricing, sourced from Esios [32]. The system optimized energy consumption and storage based on price signals.

On one hand, during hours of low electricity prices (from 14:45 p.m. to approximately 19:00 p.m.), the system focused on local consumption, ensuring that the PV energy generated and stored was utilized efficiently for the local loads. To achieve this, the setpoint was set to zero, preventing the inverter from injecting energy into the grid (clearly seen in Figure 9a). During these hours of the test, PV production was low, so the BESS compensated for the deficit.

On the other hand, when electricity prices rose, the system adopted a dual strategy of self-consumption and selling excess energy to the grid. This approach allowed for cost savings and generated additional economic benefits. This can be seen clearly at the end of the test in Figure 9a from 19:00 p.m. approximately to the end. During this period, the setpoint is set positive, and with no PV production, all injected energy comes from the BESS, causing it to discharge (reflected in Figure 9c).

Finally, during low-price nighttime hours, the system would charge the battery to prepare for subsequent high-price periods, ensuring sustained economic efficiency. This time frame is not represented in the results because we could not conduct the experiment overnight due to the need for continuous supervision, preventing us from recording data between 8:00 p.m. and 8:30 a.m. Nevertheless, the graphical analysis for this PVPC self-consumption mode provides clear evidence of these strategies and demonstrates the responsiveness of the system to market conditions, achieving an optimal balance between consumption and grid interaction.

### 4.5. Summary

The results demonstrate the ability of the proposed system to operate efficiently across both flexibility market and self-consumption modes by dynamically managing energy flows based on real-time setpoints and economic conditions. As shown in Figure 9 and Figure 10, the system effectively followed the provided setpoints, maintaining precise control over energy flows and enabling energy injection from the BESS. The graph illustrates how the system dynamically adjusted the power output of the inverter to match the desired flexibility signals. This highlights the ability of the system to respond in real time, optimizing energy usage and demonstrating robust performance in energy management.

The overall performance of the system on 11 October 2024 was captured and analyzed through graphical data, showcasing its ability to consistently achieve minimal tracking error. This robust performance reflects a strong alignment with the described strategies. During the flexibility market period, the system demonstrated high reliability and precision by closely following to the instructions provided by the emulated aggregator, ensuring that all directives were executed effectively.

In addition, the dynamic of the system response to price signals further optimized its economic performance. By maintaining minimal tracking errors and adapting seamlessly to market conditions, the system achieved its operational goals. These results underscore its potential for integration into future local network operations, where similar strategies could deliver both technical and economic benefits.

## 5. Discussion

This section addresses the practical considerations and limitations of the proposed system, particularly regarding the experimental validation scope, long-term operation, transient behavior, and scalability, as exposed by the real-world test day.

### 5.1. Representativeness of Experimental Validation

The experimental validation, conducted on 11 October 2024, serves as a crucial proof of concept for the proposed indirect control system. The choice of this day, characterized by cloudy weather, introduced multiple fluctuations in PV generation. This scenario is highly representative of conditions that challenge energy management systems, moving away from the ideal performance of sunny days and providing a robust test environment for analyzing system transients.

It is acknowledged that a broader array of tests, incorporating seasonal variations, would enhance the value of the results. However, the primary objective of this work is to demonstrate the robust and correct functioning of the indirect control mechanism, which was successfully achieved. Given that the load fluctuations were intentionally controlled and manipulated to generate significant and interesting transients for system response analysis, their variation did not depend on the specific day of the experiment.

Currently, work is underway to implement this system autonomously to enable uninterrupted operation and data collection over longer periods, which will naturally incorporate a wider range of weather and seasonal conditions.

### 5.2. Long-Duration Operation and Nighttime Cycles

The current experimental setup omitted overnight charging and discharging tests due to the need for continuous supervision. This omission, while acknowledged, does not significantly affect the primary conclusions of this work, as the objective was to demonstrate the fundamental functionality of the system, which was clearly validated during the diurnal test.

Future work is focused on addressing this limitation by implementing the system for autonomous, long-term operation. This involves developing a real-time supervision system within the ESP32 microcontroller, coupled with a remote Human–Machine Interface (HMI) based on Node-RED that acts as the aggregator. This setup will allow the ESP32 to continuously communicate registered plant data to the HMI for saving and will enable the setpoint of the aggregator to be sent remotely, allowing the system to operate unattended for extended periods, including throughout the night. Such continuous testing will be essential for future studies focused on evaluating various energy management strategies.

### 5.3. System Behavior Under Transient Fluctuations

As previously addressed in Section 4, the behavior of the system under sudden fluctuations in load and PV generation is shown in Figure 9b and Figure 10b (which display the power error). Fluctuations in PV generation were observed to not generate a significant effect on the setpoint tracking. However, abrupt load changes do cause transient setpoint tracking errors. Specifically, during abrupt step changes in load, the observed transients in tracking the setpoint of the aggregator last an average of 6 s and have a maximum power error equal to the variation in the load power. This transient increase in error before stabilization is visible in the figures.

Regarding communication failures or network latency, while not explicitly tested, it is projected that these would produce similar transient power errors in setpoint tracking if changes occur when the aggregator is sending new setpoints. The duration of the tracking error in these cases would be the same as the duration of the latency or communication failure. This is a consideration that will be addressed in future work focused on implementation robustness and scalability.

### 5.4. Scalability and Multi-Inverter Microgrids

The proposed ESP32-based EMS and its indirect control method via wattmeter emulation are specifically designed for residential PV microgrids. Then, for a larger microgrid with multiple inverters, the system would be conceived as a modular solution: each inverter would have its own dedicated ESP32-based control system. Therefore, there would be no significant change at the level of the individual system component. The necessary modification would be in the strategy of the aggregator, which would need to be designed to account for multiple inverters and send the corresponding individual setpoints to each ESP32-based controller. Thus, the core control methodology will remain viable, with the complexity shifting to the higher-level coordination logic of the aggregator.

### 5.5. Economic Benefit

Based on the tests carried out, the proposed system enables the implementation of energy arbitrage strategies and controlled power injection from both the battery and the inverter, optimizing energy exchange with the grid. Considering an average price differential in the Spanish PVPC tariff of approximately 0.15 €/kWh between off-peak and peak periods [32], and a usable battery capacity of 5.8 kWh, the approximate annual economic benefit from daily operation can be estimated as follows:

(1) AnnualEcon.Benefit=5.8kWh/day·0.15€/kWh·365days/year≅317€/year

In addition, potential revenues could be obtained from participation in local flexibility markets, where prosumers will be able to provide power adjustment and ancillary services to distribution networks. Although these mechanisms are not yet fully operational and precise data are still unavailable, it is expected that the additional remuneration could increase the total benefits by around 50% compared to the base case, reaching approximately 480–500 € per year.

### 5.6. Comparative Analysis with Advanced Flexible EMS Methodologies

Recent flexible EMS strategies proposed in high-impact literature typically prioritize algorithmic sophistication to address large-scale and stochastic problems. This focus often introduces significant cost and hardware barriers for practical residential implementation. To contextualize the contribution of our work, we compare our ESP32-based EMS against two advanced reinforcement learning (RL) approaches representing the related work in complex energy optimization:

- The algorithm of Hierarchical Reinforcement Learning (HRL) proposed by [33] for regional multi-energy markets.
- The approach of Hybrid Policy-Based Reinforcement Learning (HPRL) by [34] for adaptive energy management in transmission-constrained island groups.

While comparative benchmarks are essential for robust scientific validation, a direct, quantitative comparison of our indirect control mechanism against standard rule-based or optimization-based EMS (e.g., MILP or MDP) is fundamentally challenging. This is because these advanced systems assume direct, precise control over all power flows and typically operate on powerful hardware. In contrast, our core objective is to validate a low-cost, practical enablement mechanism (the ESP32 system taking the place of the wattmeter) that unlocks a restricted capability in commercial household inverters: the ability to inject energy from the BESS into the grid. Therefore, metrics like algorithmic efficiency or optimization depth are less relevant than focusing on flexibility enablement and real-time execution. We benchmarked the tracking performance of our system against the commanded setpoint (emulating aggregator instructions), and our results demonstrate minimal tracking errors, confirming the robust viability of the system for flexibility market participation. This achievement precedes any comparative evaluation of specific control algorithms.

Table 1 summarizes this comparison, highlighting a critical dichotomy between algorithmic depth and implementation feasibility for the existing prosumer installed base.

Therefore, while the advanced RL-EMS solutions (HRL and HPRL) are designed for the management of long-term stochastic complexity [33,34], their reliance on simulation and high computational requirements limit their immediate adoption by residential prosumers using commercial inverters. Our work addresses the most critical practical bottleneck: the hardware limitation. By implementing a pragmatic, low-cost indirect control solution validated in a real-world physical environment, our system successfully bypasses the BESS energy injection restriction imposed by commercial inverters, which is an essential step toward democratizing flexibility participation in the residential sector.

## 6. Conclusions and Future Work

This project successfully implements a flexible EMS using the ESP32 microcontroller for a PV microgrid with battery storage (or BESS). By altering the measurements of the wattmeter and leveraging indirect control over the behavior of the inverter, the system is able to effectively manipulate power flows between the microgrid and the grid without requiring direct control commands, which are often unsupported by commercially available inverters. One of the most notable contributions is the ability of the proposed system to force the inverter to inject energy from the BESS into the grid. Moreover, the innovative approach of using an artificial offset in wattmeter readings to adjust the power injection and absorption of the inverter demonstrated the viability of low-cost, real-time energy management in prosumer settings.

Through real-world experimentation, the system was able to achieve the desired level of flexibility, adjusting energy flows in response to market signals and the demands of the grid. The results validate that such an approach can optimize the usage of stored and generated energy, contributing to more efficient participation in local energy markets, peak shaving strategies, and overall demand response. However, despite the successful development and implementation, the work highlighted several areas for improvement. The reliance on indirect control methods presents some limitations in the precision of power management, and the alteration process of the readings, while functional, introduces potential complexities in scaling the system to more intricate setups or larger microgrids.

Future work will focus on several enhancements to improve the system. First, integrating direct control of inverters through APIs or protocols like MQTT would allow for more precise and responsive power management, eliminating the need for indirect methods. Additionally, testing the scalability of the system in larger microgrids or across multiple prosumer setups will be crucial to evaluate its performance in more complex environments. Also, refining the control algorithm by incorporating ML or predictive models could further optimize energy management, considering factors like weather forecasts and market conditions. Finally, integrating the system with local energy markets would enable automatic responses to real-time price signals, enhancing economic benefits for prosumers while also improving user satisfaction and system resilience under practical conditions, making it a robust solution for residential and small-scale commercial energy applications.

## Figures and Tables

**Table 1:** Comparison of flexible energy management methodologies.

Comparison Criterion	Proposed EMS	[33]	[34]
Core Methodology	Indirect Control (Wattmeter Emulation)	HRL	HPRL
Execution Platform	Low-Cost/IoT	High-Performance Computing Platform	High-Performance Computing Platform
Main Objective	Unlock BESS Injection in Commercial Inverters	Trading Optimization in Regional Market	Adaptive Management in Constrained Island Systems
Validation Environment	**Real-World Physical Testbed**	Simulation/Numerical Examples	Simulation
Entry Barrier (Cost)	Low	High	High

## Data Availability

The raw data supporting the conclusions of this article will be made available by the authors on request.

## Abbreviations

The following abbreviations are used in this manuscript:

AC: Alternating Current, AI: Artificial Intelligence, BESS: Battery Energy Storage System, CPP: Critical Peak Pricing, DC: Direct Current, DERs: Distributed Energy Resources, DR: Demand Response, DSM: Demand-Side Management, DSO: Distribution System Operator, EMS: Energy Management System, EVs: Electric Vehicles, GAs: Genetic Algorithms, GUI: Graphical User Interface, IoT: Internet of Things, MDP: Markov Decision Processes, MILP: Mixed-Integer Linear Programming, ML: Machine Learning, MPPT: Maximum Power Point Tracking, NREL: National Renewable Energy Laboratory, OMI: Iberian Market Operator, PHIL: Power Hardware-in-the-Loop, PSO: Particle Swarm Optimization, PV: Photovoltaic, PVPC: Precio Voluntario para el Pequeño Consumidor (Voluntary Price for Small Consumers), REE: Red Eléctrica Española (Spanish Electric Grid), RTP: Real-Time Pricing, SCADA: Supervisory Control and Data Acquisition, SoC: State of Charge, TCP: Transmission Control Protocol (Modbus TCP), ToU: Time-of-Use, UAH: University of Alcalá, UART: Universal Asynchronous Receiver–Transmitter, V2G: Vehicle-to-Grid, V2H: Vehicle-to-Home.
